# Inorganic Nitrogen Application Affects Both Taxonomical and Predicted Functional Structure of Wheat Rhizosphere Bacterial Communities

**DOI:** 10.3389/fmicb.2018.01074

**Published:** 2018-05-29

**Authors:** Vanessa N. Kavamura, Rifat Hayat, Ian M. Clark, Maike Rossmann, Rodrigo Mendes, Penny R. Hirsch, Tim H. Mauchline

**Affiliations:** ^1^Sustainable Agriculture Sciences, Rothamsted Research, Harpenden, United Kingdom; ^2^PMAS Arid Agriculture University, Rawalpindi, Pakistan; ^3^Laboratory of Environmental Microbiology, Embrapa Meio Ambiente, Jaguariúna, Brazil

**Keywords:** wheat, rhizosphere, bacterial communities, fertilizer, functional diversity, sustainable intensification

## Abstract

The effects of fertilizer regime on bulk soil microbial communities have been well studied, but this is not the case for the rhizosphere microbiome. The aim of this work was to assess the impact of fertilization regime on wheat rhizosphere microbiome assembly and 16S rRNA gene-predicted functions with soil from the long term Broadbalk experiment at Rothamsted Research. Soil from four N fertilization regimes (organic N, zero N, medium inorganic N and high inorganic N) was sown with seeds of Triticum aestivum cv. Cadenza. 16S rRNA gene amplicon sequencing was performed with the Illumina platform on bulk soil and rhizosphere samples of 4-week-old and flowering plants (10 weeks). Phylogenetic and 16S rRNA gene-predicted functional analyses were performed. Fertilization regime affected the structure and composition of wheat rhizosphere bacterial communities. Acidobacteria and Planctomycetes were significantly depleted in treatments receiving inorganic N, whereas the addition of high levels of inorganic N enriched members of the phylum Bacteroidetes, especially after 10 weeks. Bacterial richness and diversity decreased with inorganic nitrogen inputs and was highest after organic treatment (FYM). In general, high levels of inorganic nitrogen fertilizers negatively affect bacterial richness and diversity, leading to a less stable bacterial community structure over time, whereas, more stable bacterial communities are provided by organic amendments. 16S rRNA gene-predicted functional structure was more affected by growth stage than by fertilizer treatment, although, some functions related to energy metabolism and metabolism of terpenoids and polyketides were enriched in samples not receiving any inorganic N, whereas inorganic N addition enriched predicted functions related to metabolism of other amino acids and carbohydrates. Understanding the impact of different fertilizers on the structure and dynamics of the rhizosphere microbiome is an important step toward developing strategies for production of crops in a sustainable way.

## Introduction

The development and application of synthetic fertilizers fueled the green revolution and massively expanded our ability to produce food (Stevenson et al., 2013). However, current agricultural practices rely on unsustainable levels of inorganic nitrogen and phosphorus fertilizers, pesticides and other chemical inputs which are environmentally harmful (Rees et al., 2013; van Grinsven et al., 2013). Wheat is the third most grown cereal in the world, and the FAO predicts that by 2050 the global population will reach 9 billion meaning food production must be increased by 70% (FAO, 2009). By 2020, there will be an increased demand in this crop to 746 million tons, which is 11% above the annual production recorded between 2009 and 2010 (OECD/FAO, 2011).

It is well known that soil microbial communities influence plant growth, health and resource use efficiency, especially the subset that are recruited by plants to form the root microbiome (Berendsen et al., 2012; Mendes et al., 2013). Rhizosphere microorganisms can benefit crop plants in several ways including improved plant nutritional status and protection against biotic and abiotic stresses (Bloemberg and Lugtenberg, 2001; Mendes et al., 2013; Turner et al., 2013; Choudhary et al., 2016; Ahkami et al., 2017). Understanding and optimizing microbial communities for enhanced crop growth is a considerable challenge, but one that we can now begin to tackle due to advances in next-generation sequencing technologies (Mendes et al., 2013). An understanding of the effect of agricultural practices such as fertilization regime on the presence or absence of pathogens and beneficial microbes is essential to ascertain if microbiome manipulation can be optimized to contribute to sustainable intensification of agriculture (Bakker et al., 2012; Hartmann et al., 2015; Quiza et al., 2015).

The Broadbalk experiment at Rothamsted Research in the United Kingdom, established in 1843, is the longest running scientific experiment in the world, and is suitable to study interactions between agricultural fertilization practices, the wheat rhizosphere microbiome and wheat yields. At Broadbalk it has been demonstrated that wheat yields with the standard mineral fertilization rate of 144 kg N. ha^-1^ per year are equal to that of farmyard manure (FYM), though the soil structure and root biomass are very different (Rothamsted Research, 2006). Other previous studies at this site have focused on the effect of fertilizer treatments on nutrient cycling, as well as microbial activity in bulk soil by targeting specific genes together with metagenomic approaches (Jenkinson and Parry, 1989; Glendining et al., 1996; Knights et al., 2001; Clark and Hirsch, 2008; Clark et al., 2012; Ogilvie et al., 2008; Zhalnina et al., 2013). However, little work has investigated the effects of fertilization regime on the structure and stability of rhizosphere bacterial communities. Here, next generation Illumina 16S rRNA gene amplicon sequencing was used to assess how the rhizosphere bacterial microbiome of wheat is shaped over time in soil from the continuous wheat strips of Broadbalk which have received inorganic fertilization, organic nitrogen as farmyard manure or no N fertilizer treatment continuously for more than 170 years.

## Materials and Methods

### Soil Sampling, Experimental Setup, Harvesting and Chemical Analyses

We evaluated the rhizosphere collected from wheat plants grown under four nitrogen regimes: organic N, zero N, medium inorganic N and high inorganic N, at two time points: 4 and 10 weeks. As reference and to evaluate the rhizosphere effect, an equivalent number of bulk soil samples related to the same treatments were also collected. For that, soil was sampled in January 2013 from the Broadbalk experiment (51°48′33″ N; 0° 22′19″ W) at Rothamsted Research, Harpenden, Hertfordshire, United Kingdom, section 6 (continuous wheat plots) which did not receive spring or summer fungicides. The samples were taken before the addition of NH_4_NO_3_ fertilizer to avoid any spiking effects, although farmyard manure had been applied to the relevant plot in October of the previous year. Soil samples were taken from four plot treatments: organic nitrogen as farmyard manure (FYM) at 35 t per hectare (strip 2.2); zero nitrogen (strip 5); mineral fertilizer in the form of ammonium nitrate at rates of 144 (strip 8) and 288 (strip 16) kg per hectare per year (**Table 1**).

**Table 1:** Fertilization regime adopted in Broadbalk experiment for the four treatments used in this study.

Strips	Treatment	From 1843	From 1968	From 1985	Since 2001
2.2	Organic N	FYM	FYM	FYM	FYM
5	Zero N	PKNaMg	PK(Na)Mg	PKMg	(P)KMg
8	Medium inorganic N	N3 PKNaMg	N3 PK(Na)Mg	N3 PKMg	N3 (P)KMg
16	High inorganic N	N^∗^2 PKNaMg	N2 PK(Na)Mg	N6 PKMg	N6 (P)KMg

*Values represent the annual treatment per hectare. FYM, Farmyard manure at 35t. P, 35 kg as triple superphosphate. (P), Since 2001, P has not been applied to all strips as the levels of plant-available P are high. K, 90 kg as potassium sulfate. Na, 16 kg as sodium sulfate until 1973 and no longer applied since then (Na). Mg, 12 kg as Kieserite. Until 1973, it was 11 kg as magnesium sulfate and 35 kg every 3rd year from 1974 until 2000. N, as ammonium nitrate (Nitram, 34.5% N) since 1986; as calcium ammonium nitrate (Nitro-chalk, c.26% N) from 1968 until 1985; as ammonium sulfate or sodium nitrate (N^∗^) until 1967. N2, 96 kg as a single application. N3, 144 kg as a single application. N6, 288 kg as a single application.*

All strips apart from the FYM strip have been treated with triple superphosphate at 35 kg per hectare, potassium sulfate at 90 kg per hectare, sodium sulfate at 16 kg per hectare until 1973 and Mg as kieserite at 12 kg per hectare. Inorganic nitrogen has been annually applied since 1985 as ammonium nitrate (Nitram, 34.5%). A total of 20 kg of soil was sampled randomly across each strip using a small hand trowel which had been washed in ethanol (70%) before use and between different treatments. Soil was spread-out on to an approximate depth of 3 cm and air dried in the laboratory for 24 h. After this time the soil was sieved through a 2-mm mesh sieve, thoroughly mixed in polythene bags and used to fill 13-cm pots which were allowed to equilibrate in the glass house for 1 week at 20°C with a 16-h per day light regime. Any weeds germinating in the pots were removed by hand. *Triticum aestivum* cv. Cadenza seeds were surface sterilized with 70% ethanol for 10 min and then with 1.5% active chlorine for 1 h and subsequently rinsed 5 times in sterile distilled water prior to overnight imbibition in sterile water at 4°C prior to planting. All pots were watered daily with tap water.

**FIGURE 1:**
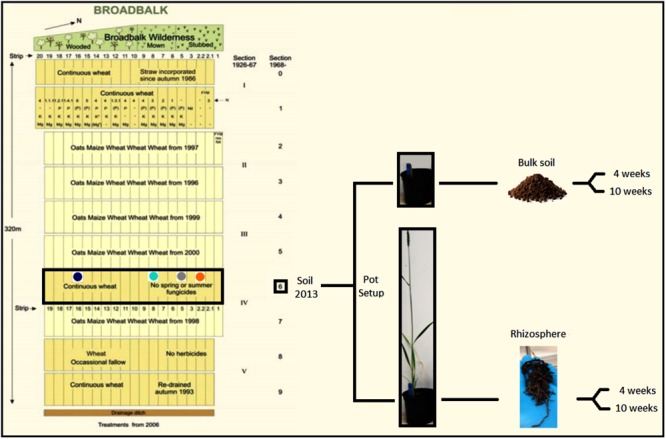
Schematic representation of the experimental setup. Soil samples were collected from the Broadbalk experiment (section 6) in 2013 from strips under different fertilization regime [2.2 = organic N (orange); 5 = zero N (gray); 8 = medium inorganic N (turquoise); 16 = high inorganic N (dark blue)]. Soil was used to grow *Triticum aestivum* cv. Cadenza in pots. Bulk soil and rhizosphere samples were collected after 4 and 10 weeks. Modified from Rothamsted Research (2006).

Three pots containing seedlings from each treatment were harvested after 4 weeks of incubation, and a further three pots were harvested at the start of flowering stage (Zadoks growth stage 61) (Zadoks et al., 1974) (after 10 weeks) for all treatments except organic N treatment which contained four pots, resulting in 25 rhizosphere samples. An equivalent number of bulk soil pots with no plant were harvested at both sampling points and this was done by firstly removing the top layer of soil with a sterile scalpel, which often was covered in algal growth, tipping the entire pot into a polythene bag, shaking and subsampling a 5-g sample. Plants were harvested by gently tipping the soil from a pot onto a fresh polythene bag. Loose soil was discarded and non-rhizosphere soil gently removed. After vigorously shaking the roots to release tightly attached soil, which was considered as rhizosphere, it was mixed to homogenize and soil was stored at -80°C prior to soil DNA extraction. For 4-week old plants around 2 g of soil was recovered, whereas flowering plants typically yielded around 5 g of rhizosphere soil. A summary of the experimental design is represented in **Figure 1**.

Soil chemical analyses were performed as described by Hirsch et al. (2017) by the Analytical Unit at Rothamsted Research on <2 mm air-dried soils. Soil pH was measured in a 10 g soil:25 mL 18MΩ deionised water suspension. Inorganic carbon (IC) was determined using a Primacs inorganic carbon analyser and N and C were determined using a LECO TruMac Combustion Analyser.

### Soil DNA Extraction and Quantification

For each sample, DNA was extracted from 0.25 g rhizosphere soil using the MoBio PowerSoil^TM^ DNA Isolation Kit (Carlsbad, CA, United States). Extractions were performed according to the manufacturer’s instructions but with the use of the MP Biomedicals FastPrep-24 machine twice for 30 s at 5.5 m.s^-1^. DNA purity and concentration were determined by NanoDrop spectrophotometry (Thermo Scientific, Wilmington, DE, United States) as well as a Qubit 2.0 Fluorimeter using ds DNA HS assay kit (Thermo Fisher), respectively.

### Illumina Bacterial 16S rRNA Gene Sequencing

The bacterial 16S rRNA gene was amplified from soil and rhizosphere DNA samples, using barcoded universal prokaryotic primers 515F (5′-GTGCCAGCMGCCGCGGTAA-3′) and 806R (5′-GGACTACHVGGGTWTCTAAT-3′) for paired-end microbial community (Caporaso et al., 2011) targeting the V4 region and subjected to Illumina^®^ sequencing using the MiSeq platform to generate 2 × 150 bp paired-end reads at the High-throughput Genome Analysis Core (HGAC), Argonne National Laboratory (Lemont, IL, United States).

### Sequence Analysis Pipeline

16S rRNA gene sequences were analyzed using the pipeline proposed by the Brazilian Microbiome Project (BMP) available at: http://brmicrobiome.org (Pylro et al., 2014) which uses Quantitative Insights Into Microbial Ecology (QIIME) (version 1.8.0) (Caporaso et al., 2010) and USEARCH 9.0 (Edgar, 2010)^1^. Operational taxonomic units (OTUs) were defined to 97% sequence identity against SILVA 128 database (Quast et al., 2012).

Significant differences in bacterial community structure were investigated by Permutational Analysis of Variance (PERMANOVA, Anderson, 2001) in Paleontological Statistics Software Package for Education and Data Analysis (PAST) (Hammer et al., 2001). PCoA plots, Mantel test, Analysis of Similarities (ANOSIM, Clarke, 1993) values were obtained using the same software. OTUs with less than 10% of their values containing less than two counts were filtered out. Relative log expression (RLE) data transformation was used, followed by DESeq2 tool which was used to evaluate differentially abundant taxa (expressed as log-transformed counts) using MicrobiomeAnalyst tool available at http://www.microbiomeanalyst.ca/faces/home.xhtml. Number of observed OTUs and diversity based on Shannon index were calculated in QIIME.

Raw reads used in this study can be found in the NCBI Sequence Read Archive (SRA) under accession number: PRJNA454003 and additional data is available from the corresponding author on reasonable request.

### Functional Prediction From 16S rRNA Gene

Tax4Fun (Aßhauer et al., 2015) which is an open-source R package was used to predict the functional capabilities of bacterial communities based on 16S rRNA gene amplicon data. An OTU table was created using a closed-reference picking protocol with SILVA 123 database (Quast et al., 2012) at 97% identity in QIIME. Functional diversity based on Shannon index was calculated according to Taketani et al. (2015), where the command alpha_diversity.py in QIIME was used on the functional table obtained with Tax4Fun, which was previously converted into biom format. The table with 16S rRNA gene-predicted functions was exported into excel and other functional categories that were not related to amino acid metabolism, carbohydrate metabolism, cell motility, energy metabolism, membrane transport and metabolism of terpenoids and polyketides were removed to focus the analyses on the main functions. Statistical Analysis of Metagenomic Profiles (STAMP) (Parks et al., 2014) was used to analyze statistical differences between two groups of samples using Welch’s *t*-test, followed by Benjamini–Hochberg-FDR as a multiple test correction (Benjamini and Hochberg, 1995).

## Results

### The Influence of Fertilizer Treatment on Bacterial Community Structure in the Bulk Soil and Rhizosphere

Mantel test indicated that relative abundance of OTUs assigned to bacterial communities correlated with soil parameters such as pH, %N, %C, %IC (inorganic carbon) and C:N ratio (*R* = 0.6328, *p* = 0.0001) (**Table 2**). Relative abundance of some phyla was positively and negatively correlated with particular parameters (**Supplementary Table S1**). Acidobacteria, Chloroflexi, Fibrobacteres, Latescibacteria, Nitrospirae, Planctomycetes, and Verrucomicrobia were all positively correlated with high pH, %IC and C:N ratio. Conversely, Actinobacteria and Proteobacteria were negatively correlated with these parameters. The phylum Bacteroidetes was negatively correlated with high %IC and C:N, Cyanobacteria was negatively correlated with high %N and %C, whereas Gemmatimonadetes was negatively correlated with high C:N. Lastly, Firmicutes was positively correlated with high %N and %C and negatively correlated with high pH.

**Table 2:** Values obtained from soil chemical analyses.

Treatment	pH	%N	%C	%IC	C:N
Organic N	7.27	0.295	3.21	0.04	10.88136
Zero N	7.57	0.096	0.98	0.04	10.20833
Medium inorganic N	6.35	0.116	1.06	0.02	9.137931
High inorganic N	5.74	0.161	1.14	0.01	7.080745

**FIGURE 2:**
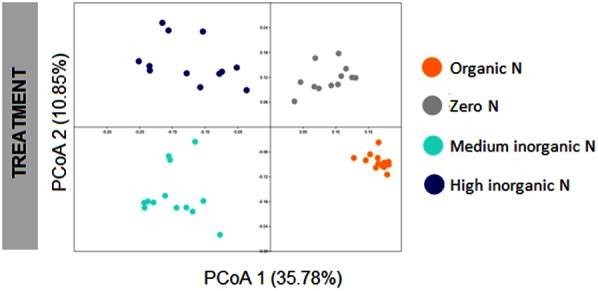
PCoA plot based on Bray-Curtis distance matrix of bacterial communities associated with wheat grown under different fertilization regime (organic N, zero N, medium inorganic N, and high inorganic N), showing treatment as the main factor driving the structure of bacterial communities for bulk soil and rhizosphere samples collected at two stages: 4 and 10 weeks. The percentage shown in each axis corresponds to the proportion of variation explained.

To assess the structure of bacterial communities associated with wheat plants grown in soils with different fertilization regimes collected at two stages, from both bulk soil and rhizosphere, OTU abundances were used to compute Bray-Curtis similarity matrix, which was used to perform principal coordinate analysis (PCoA). Bacterial communities could be discriminated according to fertilization regime (ANOSIM, *R* = 0.812, *p* < 0.001), with four main clusters clearly observed (**Figure 2**). The first axis, which corresponds to 35.78% of the variation, separated samples based on the use of inorganic nitrogen, with samples on the left representing those which received medium and high levels of inorganic nitrogen and samples on the right being those which did not receive any inorganic nitrogen inputs.

**FIGURE 3:**
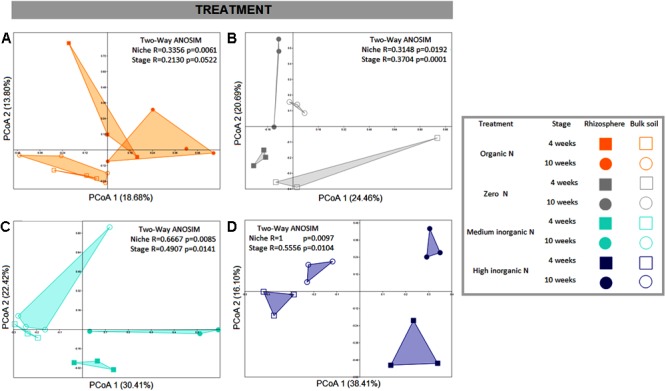
PCoA plots based on Bray-Curtis distance matrix of bacterial communities associated with wheat grown in soils under different fertilization regime. Each fertilizer treatment is represented in one PCoA: **(A)** – organic N; **(B)** – zero N; **(C)** – medium inorganic N; **(D)** – high inorganic N. The percentage shown in each axis corresponds to the proportion of variation explained. Solid squares represent rhizosphere collected after 4 weeks; solid circles represent rhizosphere samples collected after 10 weeks; open squares indicate bulk soil samples collected after 4 weeks and open circles indicate samples collected after 10 weeks.

PCoAs were also generated for each fertilization regime, to obtain a higher resolution analysis of bacterial community structure. For all fertilization regimes bulk and rhizosphere soil communities were distinguishable (**Figure 3**) and two-way ANOSIM values for each treatment corroborate that. However, the addition of inorganic nitrogen fertilizer resulted in the strongest discrimination of sample types and the application of the highest amount of inorganic nitrogen fertilizer resulted in the most pronounced temporal community shifts for both rhizosphere and bulk soil samples (**Figures 3C,D**).

### Bacterial Richness, Diversity and Functional Diversity in the Rhizosphere

We further analyzed rhizosphere samples to determine the drivers of these shifts in terms of abundance, richness and diversity of bacterial taxa as well as bacterial functional diversity (**Table 3**). Bacterial richness (observed OTUs) was highest for strips receiving farmyard manure regardless of sampling time and decreased significantly with the addition of inorganic nitrogen, the lowest recorded OTU complexity being for rhizosphere samples from inorganic N-treated soil of 10-week-old plants. Rhizosphere samples derived from zero N strip had lower bacterial species richness than the equivalent samples from organic-treated soil, but greater species richness than rhizospheres derived from strips which had received inorganic N. For all samples with the exception of those cultured in FYM treated soil, there was a decrease in bacterial species richness in the rhizospheres of 10 week relative to 4-week-old plants.

**Table 3:** Data are presented as means ± standard errors calculated for observed OTUs and Shannon diversity and functional indexes of rhizosphere bacterial communities associated with wheat grown in soils under different fertilization regimes (organic N, zero N, medium inorganic N, and high inorganic N), collected after 4 and 10 weeks.

Treatment	Stage	Observed OTUs	Shannon taxonomical diversity	Shannon functional diversity
Organic N	4 weeks	1881.5 ± 8.0 a	9.496 ± 0.010 a	10.877 ± 0.019 a
	10 weeks	1844.8 ± 5.6 a	9.502 ± 0.009 a	10.868 ± 0.012 a
Zero N	4 weeks	1691.1 ± 14.4 b	9.243 ± 0.040 a	10.892 ± 0.019 a
	10 weeks	1546.7 ± 25.5 c	8.906 ± 0.077 b	10.872 ± 0.015 a
Medium inorganic N	4 weeks	1332.7 ± 22.4 d	8.458 ± 0.075 c	10.892 ± 0.007 a
	10 weeks	1102.1 ± 23.3 f	7.543 ± 0.144 d	10.932 ± 0.051 a
High inorganic N	4 weeks	1253.8 ± 11.8 e	8.193 ± 0.034 c	10.908 ± 0.001 a
	10 weeks	1170.2 ± 3.5 f	8.208 ± 0.021 c	10.871 ± 0.009 a

*Different letters for each column denote statistical differences as indicated by Tukey test (*p* < 0.05).*

Bacterial taxonomical diversity was highest for rhizosphere samples derived from soil receiving farmyard manure, regardless of sampling time as well as for rhizospheres of 4-week-old plants grown in zero N-treated soil. The bacterial diversity for zero N treatment significantly decreased with time. Rhizosphere samples derived from inorganic N-treated soil had the lowest bacterial taxonomical diversity. In contrast to taxonomical diversity, bacterial functional diversity did not vary significantly across different treatments or sampling times.

**FIGURE 4:**
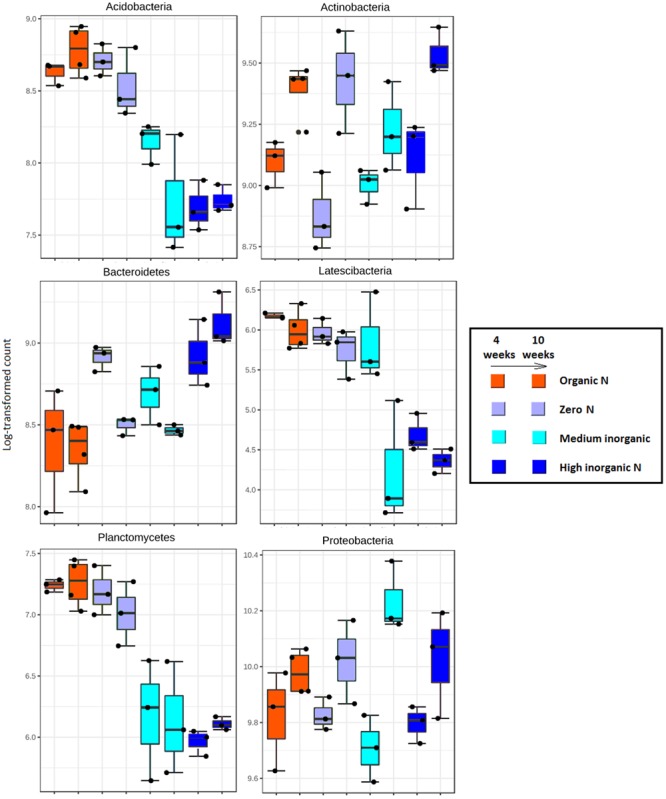
Extended error plots showing the proportion of sequences (%) that were statistically different (*p* < 0.01) for the major bacterial phyla (relative abundance > 1% for most of the samples) obtained from rhizosphere of wheat grown in soils under different fertilization regime (organic N, zero N, medium inorganic N, high inorganic N), collected at two stages: 4 weeks and 10 weeks. Each treatment is represented by bars with the same colors and they occur in pairs, with samples collected after 4 weeks on the left and samples collected after 10 weeks on the right.

Log-transformed counts obtained with DESeq2 of the dominant bacterial taxa in the rhizosphere varied over time as well as in accordance with soil fertilization regime (**Figure 4**). Sequences assigned to Acidobacteria, Latescibacteria and Planctomycetes were significantly more abundant (*p* < 0.01) in rhizosphere samples where soil had received farmyard manure or zero N inputs regardless of sampling time. Generally, inorganic N application resulted in a significant reduction in abundance of these groups in the rhizosphere. The reverse trend was found for members from the phylum Bacteroidetes as these were the most abundant in response to the highest level of fertilizer, especially after 10 weeks. Members of Actinobacteria and Proteobacteria showed an increased abundance over time, regardless of fertilization regime. Further analyses focused on genus level shifts in specific taxa. With regard to nitrogen cycling, only the ammonia oxidizing *Nitrosospira* were found to be significantly (*p* < 0.05) more abundant in samples receiving inorganic nitrogen. Thirty-three genera were found to be clearly more abundant in the treatment receiving organic nitrogen and they include members of Firmicutes (66.7%), Actinobacteria (21.2%) and Proteobacteria (12.1%). Among the phylum Firmicutes, many genera belonging to the order Clostridiales (*Caldicoprobacter, Peptoclostridium, Garciella, Mobilitalea, Dethiobacter*, and *Clostridium*) and the order Bacillales (*Planifilum, Thermobacillus, Lysinibacillus, Caldalkalibacillus, Thermoflavimicrobium, Sinibacillus*, and *Brevibacillus*) were more abundant in organic-N treated samples.

Further analyses were performed by comparing treatments with zero N with the highest inorganic N level. Thirty-four genera were enriched in the treatment receiving zero N (*p* < 0.05), and they included members of Proteobacteria (29.41%), Bacteroidetes (23.53%), Acidobacteria (14.70%), unknown (14.70%), Verrucomicrobia (8.82%), Planctomycetes (5.88%) and Nitrospirae (2.94%) (**Table 4**). Conversely, fifteen genera were enriched in treatment receiving the highest inorganic N and they include members of Proteobacteria (40%), Actinobacteria (26.67%), Bacteroidetes (20%), Acidobacteria (6.67%) and Chloroflexi (6.67%) (**Table 4**).

**Table 4:** Differentially abundant genera which were found to be enriched in samples not receiving any N and samples receiving high inorganic N.

	Phyla	Genera
Enriched in zero N	Proteobacteria	*Haliangium, Rhodobacter, Phaselicystis, Azohydromonas, Rubellimicrobium, Chelatococcus, Coxiella, Rhizobacter, Pseudofulvimonas, Panacagrimonas*
	Bacteroidetes	*Terrimonas, Lacibacter, Ferruginibacter, Dinghuibacter, Chitinophaga, Rhodocytophaga, Dyadobacter, Adhaeribacter*
	Acidobacteria	*Paludibaculum, Candidatus Solibacter, uncultured Acidobacteria, Stenotrophobacter, Vicinamibacter*
	Verrucomicrobia	*Candidatus Xiphinematobacter, Luteolibacter, Prosthecobacter*
	Planctomycetes	*Pirellula, Gemmata*
	Nitrospirae	*Nitrospira*
Enriched in high inorganic N	Proteobacteria	*Nitrosospira, Rhodanobacter, Afipia, Reyranella, Rhodovastum, Dokdonella*
	Actinobacteria	*Angustibacter, Promicromonospora, Amycolatopsis, Actinophytocola*
	Bacteroidetes	*Filimonas, Flavisolibacter, Segetibacter*
	Acidobacteria	*Granulicella*
	Chloroflexi	Uncultured *Chloroflexi*

### Diversity of Rhizosphere Bacterial Communities Over Time

**FIGURE 5:**
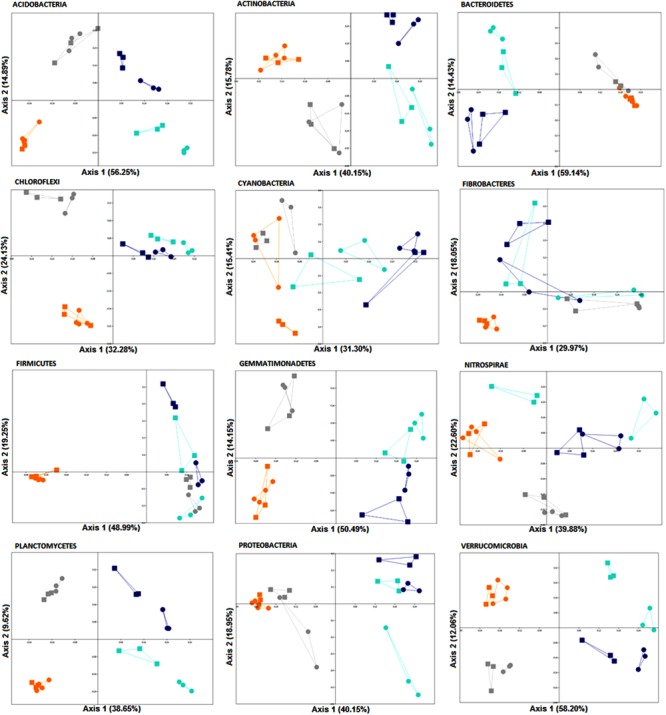
PCoA plots generated for each phylum based on Bray-Curtis distance of rhizosphere bacterial communities associated with wheat grown in soils under different fertilization regime (orange = organic N, gray = zero N, turquoise = medium inorganic N, and dark blue = high inorganic N), collected at two stages (4 and 10 weeks). The percentage shown in each axis corresponds to the proportion of variation explained. Squares represent samples collected after 4 weeks and circles represent samples collected after 10 weeks.

A two-way PERMANOVA analysis was used to test the effects of fertilization treatment (organic N, zero N, medium inorganic N, and high inorganic N) and stage (4 and 10 weeks) on the bacterial community structure at the phylum level. Based on the obtained F values, for all the analyzed phyla, except for Fibrobacteres, the effect of fertilization regime was higher (*p* = 0.0001) than growth stage effect and both factors were significantly higher than their interaction (**Table 5** and **Figure 5**), agreeing with the community level findings in **Figure 2**.

**Table 5:** Values obtained for Two-Way PERMANOVA for each phylum calculated to check the importance of fertilization regime (organic N, zero N, medium inorganic N and high inorganic N), sampling stage (4 and 10 weeks) and their interaction on the structure of rhizosphere bacterial communities.

Phylum	Fertilization regime	Sampling stage	Interaction
Acidobacteria	*F* = 27.666 ^∗^	*F* = 5.0188 ^∗∗^	*F* = 2.1544 ^∗∗∗^
Actinobacteria	*F* = 14.048 ^∗^	*F* = 2.5747 ^∗∗∗^	*F* = 1.0036, NS
Bacteroidetes	*F* = 23.983 ^∗^	*F* = 2.9519 ^∗∗∗^	*F* = 1.4051, NS
Chloroflexi	*F* = 12.771 ^∗^	*F* = 3.1143 ^∗∗^	*F* = 1.0552, NS
Cyanobacteria	*F* = 6.1595 ^∗^	*F* = 3.626 ^∗∗^	*F* = 1.25 ^∗∗∗^
Fibrobacteres	*F* = 3.7644 ^∗∗∗^	*F* = 5.6292 ^∗∗^	*F* = 3.3804 ^∗∗∗^
Firmicutes	*F* = 16.834 ^∗^	*F* = 7.4063 ^∗^	*F* = 2.6178 ^∗∗^
Gemmatimonadetes	*F* = 22.974 ^∗^	*F* = 3.7472 ^∗∗∗^	*F* = 1.0744, NS
Nitrospirae	*F* = 13.75 ^∗^	*F* = 3.5968 ^∗∗^	*F* = 2.6141 ^∗∗^
Planctomycetes	*F* = 8.6075 ^∗^	*F* = 2.5697 ^∗∗∗^	*F* = 1.4206 ^∗∗∗^
Proteobacteria	*F* = 13.046 ^∗^	*F* = 6.2682 ^∗^	*F* = 1.855 ^∗∗∗^
Verrucomicrobia	*F* = 22.375 ^∗^	*F* = 3.7255 ^∗∗∗^	*F* = 1.9358 ^∗∗∗^

*NS, not significant. Statistical differences are marked with ^∗^ (*p* < 0.001), ^∗∗^ (*p* < 0.01), and ^∗∗∗^ (*p* < 0.05).*

Furthermore, in response to farmyard manure application (organic N treatment), rhizosphere bacterial communities from all phyla, except for Cyanobacteria, were more closely clustered, demonstrating less variation over time. Additionally, when receiving farmyard manure, composition of the phylum Firmicutes is distinct from the other treatments, indicating that the amendment has created conditions favoring the growth of resident Firmicutes, or that there is a direct input of Firmicutes into the soil from the manure. When inorganic nitrogen was applied, a clear distinction across two sampling stages of members from all phyla, except for Chloroflexi and Fibrobacteres, was observed, suggesting a decrease in community stability over time.

### 16S rRNA Gene-Predicted Functional Composition in the Rhizosphere

**FIGURE 6:**
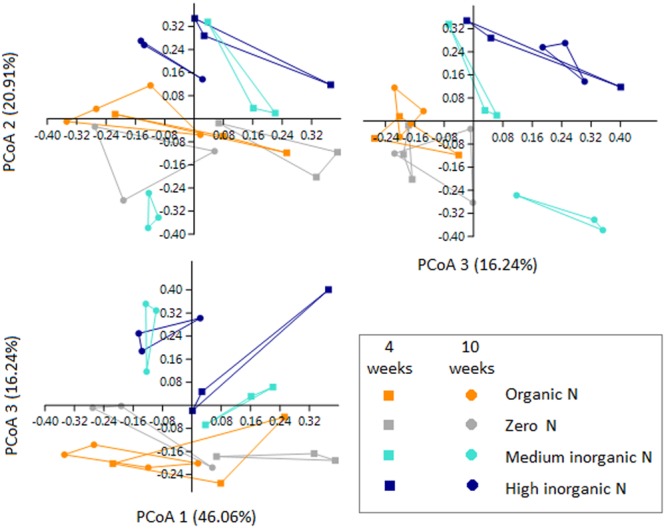
PCoA plots based on 16S rRNA gene-predicted functions for rhizosphere bacterial communities of wheat grown in soils under different fertilization (orange = organic N, gray = zero N, turquoise = medium inorganic N, and dark blue = high inorganic N), collected at two stages (4 and 10 weeks). Squares represent samples collected after 4 weeks and circles represent samples collected after 10 weeks.

**FIGURE 7:**
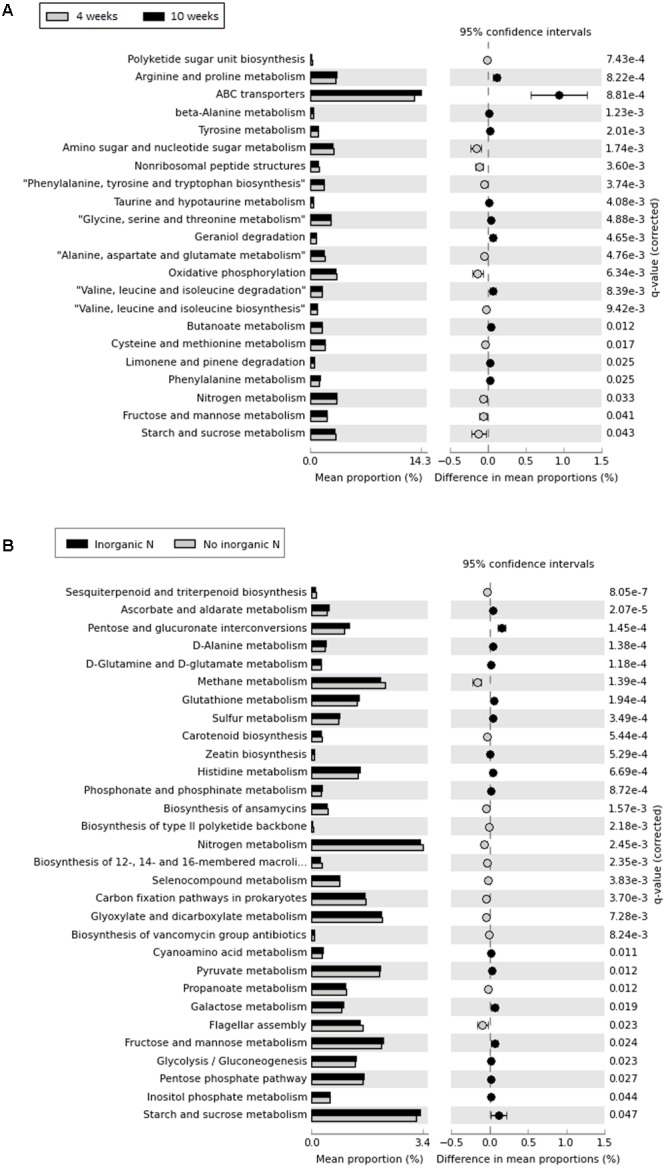
Extended error bars showing statistical differences of 16S rRNA gene-predicted functional profiles obtained with Tax4Fun. **(A)** – comparison between two developmental stage (rhizosphere collected after 4 weeks and rhizosphere collected after 10 weeks); **(B)** – comparison between addition of inorganic N, with “no inorganic N” group represented by rhizosphere samples from organic N and zero N treatments and “inorganic N,” representing rhizosphere samples from both medium and high inorganic N treatments. Corrected *p*-values were calculated using Benjamini–Hochberg FDR correction (*p* < 0.05).

Although 16S rRNA gene-predicted functional alpha diversity based on Shannon index did not vary significantly as previously shown, growth stage had major effects on the structural composition of predicted functions than fertilization regime (Two-Way PERMANOVA, Stage: *F* = 13.061, *p* = 0.0001; Treatment: *F* = 4.4602, *p* = 0.0002) (**Figure 6**). The first axis corresponding to 46.06% of the variation, shows a trend toward separation of samples according to growth stage, with few exceptions. From 67 KEGG ortholog pathways, 33% of predicted functions were significantly (*p* < 0.05) impacted by different growth stage. Extended error bar plots show statistical differences of pathways that include amino acid metabolism, carbohydrate metabolism, energy metabolism, metabolism of terpenoids and polyketides and membrane transport. Young seedlings showed a higher abundance of predicted functions related to energy metabolism, whereas ABC transporters are much more abundant in the rhizosphere of older plants (**Figure 7A**). The addition of inorganic N affected predicted functional structure of rhizosphere samples, which became evident when looking at the 3rd axis of the PCoA plot (**Figure 6**). It is possible to separate samples into two groups: one group categorized as “no inorganic N” which includes samples from organic N and zero N treatments and “inorganic N” group, which includes samples from both medium and high inorganic N treatments and the 16S rRNA gene-predicted functional pathways responsible for these differences are shown in **Figure 7B**. In general, rhizosphere samples not receiving any sources of inorganic N were enriched in predicted functions related to the metabolism of terpenoids and polyketides and energy metabolism, such as the pathways of methane and nitrogen metabolism. On the other hand, rhizosphere samples receiving both medium and high levels of inorganic N were enriched in predicted functions related to metabolism of other amino acids and carbohydrates.

## Discussion

In this study, we investigated the response of wheat bacterial communities to a variety of N fertilization regimes in a pot experiment with soil from the Rothamsted Broadbalk experiment. Creating microcosms avoided external abiotic factors often encountered in field trials such as waterlogging and frost, and allowed focus of analyses on plant–microbe interactions.

The results clearly show that fertilizer treatment influenced bulk and wheat rhizosphere soil bacterial community structure. It is well known that different fertilization practices have a strong influence on the structure of bulk soil microbial communities (Chen et al., 2016; Ding et al., 2016; Francioli et al., 2016; Soman et al., 2017). However, until now, little was known about their effect on the wheat rhizosphere community and associated 16S rRNA gene-predicted functional diversity.

When analyzing each fertilization treatment separately, temporal shifts in community structure were more pronounced in the rhizosphere, than in the bulk soil, the implication being that there is a temporal selection of the rhizosphere microbiome, though it is unclear if this is driven by the plant or microbial community. A smaller temporal effect in the bulk soil was also observed in all treatments which can be explained by community changes in response to fertilizer metabolism. The FYM soil and rhizosphere samples were very similar for both time points indicating that organic amendment provided the most stable system. This agrees with Tkacz et al. (2015), who stated that microbiome community diversity and stability are enhanced with organic matter application to soil. This might be due to the improved soil properties, nutrients and structure (Karami et al., 2012; Ai et al., 2015), or through addition of persistent spore forming Firmicutes to soil. Indeed, bacterial community structure in soils amended with farmyard manure were more distinct from non-amended soil (zero N), as well as soil treated with inorganic nitrogen and this observation agrees with other studies (Sun et al., 2004; Ai et al., 2015).

The bulk soil at this site has previously been assessed (Zhalnina et al., 2013), and so we chose to focus on rhizosphere samples in this study. It is widely known that several factors influence rhizosphere bacterial community structure, such as soil type, plant species, season, climate, agricultural management and practices (Buée et al., 2009). Rhizosphere bacterial communities receiving different N treatments were clearly distinct from each other, however, it is not possible to assign these changes to direct or indirect effects of long-term fertilization practices or a combination of both. Fertilizers can directly influence the rhizosphere microbiome through the provision of nutrients, however indirect effects are also likely to be important and include alterations of soil characteristics such as pH (Zhang et al., 2017) and the plant’s response to fertilizer application via adjusted root architecture, which could provide altered microbial niche colonization sites (Geisseler and Scow, 2014). Modulation of the rhizosphere community structure might also be due to fertilizer-induced changes in composition and/or quantity of root exudates (Zhu et al., 2016). The influence of fertilization regime on exudate profiles is poorly understood though, for a limited number of crops it has been demonstrated that exudation can be increased under nitrogen fertilization (Zhu et al., 2016; Ge et al., 2017) and that exudate composition can vary quantitatively and qualitatively in line with the nutritional status of plants (Carvalhais et al., 2011). It has been suggested that root exudates can alter microbial nitrogen fixation and nitrification pathways (Coskun et al., 2017a) and inhibit nitrification processes (Subbarao et al., 2015). It is well established that some compounds are imperative for legume-rhizobia symbiosis (Menéndez et al., 2017) and arbuscular mycorrhizal associations with plants (Basu et al., 2018) and although these examples are for secondary metabolite-mediated communication between some plant hosts and specific members of the soil microbiome, application of high levels of nitrogen fertilizers negatively influence their establishment (Grillo et al., 2016; Verzeaux et al., 2017). It is possible that subtle, as yet not understood, beneficial interactions between the microbiome and non-leguminous plants are suppressed under high fertilization conditions. This question could be partly addressed by studying microbial networks. The addition of organic amendments enhances the ratio of positive to negative correlations, compared with soil receiving chemical nitrogen fertilization (Xun et al., 2017), and also increases network complexity in bulk soil (Schmid et al., 2017) as well as positive interactions among fungal soil communities (Xue et al., 2018). However, little is known about the effect of inorganic nitrogen application on bacterial networks in the rhizosphere of non-leguminous plants and this should be tackled in future studies.

Rhizosphere soils amended with organic nitrogen showed the highest bacterial richness and diversity, followed by samples from zero N strip. It is unclear to what extent the FYM treatment supplements soil with a microbial source, and so direct comparisons between inorganic and organic nitrogen applications should be made with caution. However, inorganic N application was inversely proportional to microbial diversity and richness – the lowest richness and diversity indexes were observed for samples receiving inorganic N. This is in agreement with other studies (Campbell et al., 2010; Zeng et al., 2016). Additionally, with the exception of FYM-treated soil, rhizosphere bacterial species richness declined over time. Increased richness and diversity when organic amendments are used is a common trend for bulk soils (Francioli et al., 2016; Lupatini et al., 2017; Wang et al., 2017) and has been observed in the rhizosphere of other crops (Höflich et al., 2000; Lavecchia et al., 2015). This observation might be due to the improvement of soil porosity and aggregation, provided by the organic treatment, amending the soil structure (Pagliai et al., 2004). Furthermore, in organic-treated soils, microbial communities with increased diversity might be able to transform organic carbon into biomass, enriching the abundance of microbes (Mäder et al., 2002). It is not surprising that farmyard manure-treated soil showed the highest species richness because of the extra organic matter which was added to the system and due to the additional microbes provided by this treatment (Francioli et al., 2016).

Rhizosphere bacterial communities were highly responsive to different fertilizers, but such responses differed among phyla. A decrease in the abundance of members belonging to Acidobacteria, Latescibacteria, and Planctomycetes was observed in rhizospheres receiving inorganic N fertilizer and these phyla can be ecologically categorized as oligotrophic (Fierer et al., 2007). On the other hand, copiotrophic group including the Bacteroidetes was more abundant with addition of high levels of inorganic N. Ramirez et al. (2012) observed lower microbial respiration rates in soils amended with nitrogen, hypothesizing that N addition suppresses soil microbial activity by shifting bacterial metabolism. As a response, communities more capable of degrading labile compounds (copiotrophic) increased as opposed to oligotrophic bacteria.

When focusing the analyses on specific taxa, several genera belonging to Firmicutes and Actinobacteria were found to be more abundant in the treatment receiving organic nitrogen. Further comparisons were made between zero nitrogen and high inorganic nitrogen treatments and some genera were found to be enriched in samples not receiving inorganic nitrogen. **Table 4** highlights the differences in relative abundance of specific OTUs found under N limited and replete conditions. Although it is not possible to assign plant growth promotion function by 16S rRNA gene sequence, a number of bacterial genera which are enriched in the zero-fertilizer treatment, have previously been shown to benefit plants. For example, *Rhodobacter* spp. has plant growth promotion potential and *Haliangium* spp. are putative biocontrol agents (Fudou et al., 2001). Additionally, *Dyadobacter* spp. have been shown to solubilise phosphorous (Pandya et al., 2015) and to have affinity for wheat (Tkacz and Poole, 2015). In addition, other OTUs were enriched in this environment from phyla not usually associated with rhizosphere colonization representative of the Verrucomicrobia and Planctomycetes. As such, the improvement of culturing methods for oligotrophic microorganisms and their subsequent bioprospecting for PGPR traits could be one unexplored resource for the development of new inoculants.

Functional diversity, as measured with the Shannon index, was not significantly affected by fertilizer treatment or plant growth stage. To date, most studies related to microbial functional diversity are based on methods for determining the community level physiological profile (CLPP), and in these studies, functional diversity in bulk soil and rhizosphere soil was affected by fertilization treatment (Sradnick et al., 2013; Yu et al., 2015; Shen et al., 2016). There are still very few studies using a tool like Tax4Fun to predict functions using 16S rRNA gene and to obtain functional diversity index. Tax4Fun predicts the functional or metabolic capabilities of microbial communities based on 16S rRNA gene data, providing an approximation of functional profiles obtained from metagenomic sequencing approaches. It cannot replace whole metagenomic profiling but it is a useful tool to supplement 16S rRNA gene analysis in metagenome pre-studies. Unlike PICRUSt (Langille et al., 2013), which depends on the topology of the tree and the distance to the next sequenced organism, it uses the nearest neighbor identification based on a minimum 16S rRNA gene sequence similarity. We found it a useful tool, however, caution must be taken when interpreting these results. The 16S rRNA-predicted functional diversity obtained with the Shannon index alone could be misleading, because it considered the functional composition within each sample. When we analyzed the functional structure, i.e., the diversity between different samples, some differences could be observed. Most of the functions enriched during early stages were related to energy metabolism and they might suggest a higher metabolic bacterial activity, whereas after 10 weeks, functions related to degradation of more complex compounds such as the root volatiles geraniol and limonene (Wenke et al., 2010) degradation pathways are higher, suggesting a change in the quality of root exudates over time (Houlden et al., 2008) as well as quantity as observed by the higher abundance of predicted functions related to ABC transporters, which are responsible for the exchange of compounds from the cells (Badri and Vivanco, 2009). Depletion of inorganic nitrogen mainly affected pathways related to metabolism of terpenoids and polyketides and energy metabolism, such as nitrogen and methane metabolism. Under limited N conditions, plants tend to increase the exudation of terpenoids, which are known as nitrification inhibitors (Coskun et al., 2017b). In bacteria, genes encoding terpene synthases were mostly related to production of secondary metabolites (Yamada et al., 2015), but it has been shown that monoterpenes inhibited methane oxidation by methanotrophs and denitrification by environmental isolates (Amaral et al., 1998), however, the roles of terpenoids in N cycle should be further investigated. The production of antimicrobial compounds is the most studied mechanism of bacterial competition (Hibbing et al., 2010) and the higher abundance of predicted functions related to the production of classes of antibiotics might indicate a high competitive environment, as nutrients are less available and number of bacterial species is also higher. Future experiments should compare these results with metagenomics and metatranscriptomics. We had assumed that FYM application would have resulted in a higher functional diversity due to the more varied range of substrates and an increased species richness in this treatment. This implies that there is a greater amount of microbial functional redundancy (Taketani et al., 2015; Berga et al., 2017) in the FYM treated strip. Future work should determine whether or not this is the case through metagenomics as well as microbial isolate sequencing efforts.

This study demonstrates the impact of different long-term fertilization treatments on wheat rhizosphere bacterial communities and supports previous studies showing that prolonged treatments over many years affect the bulk soil community structure. High levels of inorganic nitrogen fertilizers negatively affect bacterial richness and diversity, leading to a less stable bacterial community structure over time, whereas, more stable bacterial communities are provided by organic amendments. The plant microbiome is vitally important for plant health, and it follows that understanding the effects of different fertilizer applications on the structure and dynamics of the rhizosphere microbiome of plants is a vital first step for the sustainable production of crops in next-generation agriculture (Schlaeppi and Bulgarelli, 2015).

## Author Contributions

IC, PH, and TM: designed the experiment. RH, IC, and TM: obtained and processed the samples. VK and TM: analyzed the data. VK and TM with contribution of all co-authors: wrote the paper.

## Conflict of Interest Statement

The authors declare that the research was conducted in the absence of any commercial or financial relationships that could be construed as a potential conflict of interest.

## Footnotes

1 http://www.drive5.com/usearch

## Supplementary Material

TABLE S1 Spearman’s rank-order correlation of relative abundance of some phyla and soil parameters. Significant correlations (*p* < 0.05) are highlighted in gray, showing positive (green) and negative (red) correlations.
